# Hormone receptor mRNA and protein levels as predictors of premenopausal tamoxifen benefit

**DOI:** 10.2340/1651-226X.2024.19655

**Published:** 2024-04-08

**Authors:** Terese Engström, Maria Ekholm, Mårten Fernö, Christine Lundgren, Bo Nordenskjöld, Olle Stål, Pär-Ola Bendahl, Julia Tutzauer, Lisa Rydén

**Affiliations:** a Department of Clinical Sciences Lund, Division of Oncology, Lund University, Lund, Sweden; b Department of Oncology, Ryhov Hospital, Jönköping, Sweden; c Department of Biomedical and Clinical Sciences, Division of Oncology, Linköping University, Linköping, Sweden; d Department of Clinical Sciences Lund, Division of Surgery and Oncology, Lund University, Lund, Sweden; e Department of Surgery, Skåne University Hospital, Malmö, Sweden

**Keywords:** breast cancer, tamoxifen, premenopausal patients, randomized trial, estrogen receptor status, progesterone receptor status, predictive information

## Abstract

**Background and purpose:**

Tamoxifen remains an important adjuvant treatment in premenopausal patients with hormone receptor-positive breast cancer. Thus, determination of hormone receptors is important. Here, we compare cytosol-based methods, immunohistochemistry (IHC), and gene expression (GEX) analysis for determining hormone receptor status in premenopausal breast cancer patients from a randomised tamoxifen trial, to evaluate their performance in identifying patients that benefit from tamoxifen.

**Patients and Methods:**

Premenopausal patients (*n*=564) were randomised to 2 years of tamoxifen or no systemic treatment. Estrogen receptor (ER) and progesterone receptor (PR) status by protein expression measured by cytosol-based methods and IHC, and mRNA by GEX analysis were compared in 313 patients with available data from all methods. Kaplan Meier estimates and Cox regression were used to evaluate the treatment-predictive value for recurrence-free interval (RFi) and overall survival (OS). Median follow-up for event-free patients was 26 (RFi) and 33 (OS) years.

**Results:**

The mRNA data of *ESR1* and *PGR* distributed bimodally, patterns confirmed in an independent cohort. *Kappa*-values between all methods were 0.76 and 0.79 for ER and PR, respectively. Tamoxifen improved RFi in patients with ER-positive (ER+) or PR-positive (PR+) tumours (Hazard Ratio [HR] and 95% confidence interval [CI]), cytosol-ER+ 0.53 [0.36–0.79]; IHC-ER+ 0.55 [0.38–0.79]; GEX-ER+ 0.54 [0.37–0.77]; cytosol-PR+ 0.49 [0.34–0.72]; IHC-PR+ 0.58 [0.40–0.85]; GEX-PR+ 0.55 [0.38–0.80]). Results were similar for OS.

**Interpretation:**

These methods can all identify patients that benefit from 2 years of tamoxifen with equal performance, indicating that GEX data might be used to guide adjuvant tamoxifen therapy.

## Introduction

Premenopausal patients have much to gain in life expectancy by evading breast cancer-related death. For premenopausal women with hormone receptor-positive disease, tamoxifen is still an important adjuvant treatment option, alone or as chemo-endocrine therapy [1]. Side effects associated with tamoxifen therapy include menopausal-like symptoms, uterine cancer, and venous thromboembolism, making validation of measurements of predictive factors important to avoid recommendation of tamoxifen to patients who do not benefit from therapy [2]. The estrogen receptor (ER) is well known to be predictive of tamoxifen response, but the additional predictive value of determining progesterone receptor (PR) status, in addition to ER status, has been questioned [3].

Different methods can be used to determine hormone receptor status for tamoxifen prediction. Immunohistochemistry (IHC) has been demonstrated to be as good as, or superior to the previously used cytosol-based methods in predicting tamoxifen responsiveness [4]. The utility of *ESR1* mRNA levels for tamoxifen prediction is unclear. It has been proposed that high *ESR1* mRNA associates with greater tamoxifen benefit and that low *ESR1* mRNA associates with tamoxifen resistance [5]. Furthermore, mutations in the *ESR1* gene have been associated with endocrine resistance [6]. *PGR* mRNA levels are suggested to lack predictive value [5]. Today, gene expression (GEX) analyses are recommended for patients with an ambiguous risk of recurrences, for example, Endopredict^®^, MammaPrint^®^, Oncotype DX^®^, or Prosigna^®^. However, their predictive value is still unknown [7–9]. In these patients, GEX of *ESR1* and *PGR* could potentially be available for prediction of tamoxifen benefit.

In this study, we compare three methods for determining hormone receptor status in relation to recurrence-free interval (RFi) and overall survival (OS) in premenopausal breast cancer patients treated with tamoxifen compared to a control group in a randomised trial with long-term follow-up. The aim was to investigate if any of the three methods; protein expression by cytosol-based methods or IHC, and mRNA levels by GEX analysis, is superior at identifying premenopausal primary breast cancer patients that will benefit from 2 years of tamoxifen.

## Patients and methods

### Study population

**Figure 1:**
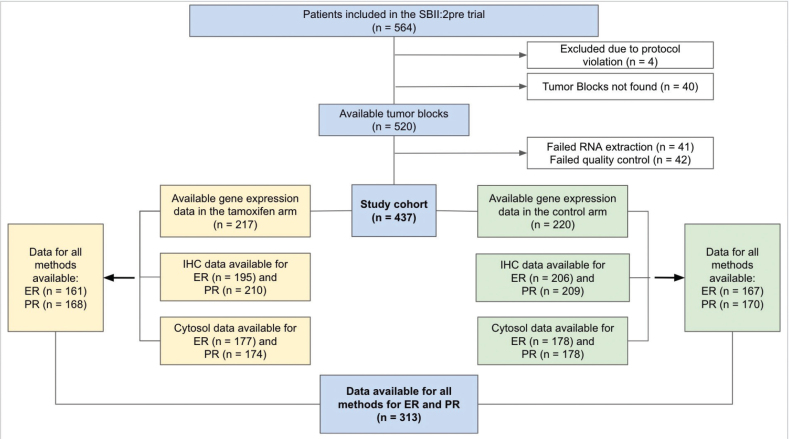
Inclusion flowchart of the study. Cytosol: cytosol-based method; ER: Estrogen receptor; IHC: immunohistochemistry; n: number of patients; PR: Progesterone receptor.

Patients randomised between 2 years of tamoxifen or no systemic treatment during 1984–1991 in the SBII:2pre trial were included (*n* = 564). This multicentre trial with two coordinating centres included premenopausal (defined as having < 1 year since last menstruation) patients with stage II (Tumor, Node, Metastasis; TNM staging system, third edition) invasive breast cancer [10]. The trial was registered in the ISRCTN database retrospectively on 6/12-2019 (https://doi.org/10.1186/ISRCTN12474687). Study population details are available in previous publications [10–13]. An inclusion flowchart for the present study is demonstrated in Figure 1.

### Validation cohort

Validation of the concept of distribution-based GEX cut-offs was performed using an independent cohort of patients with metastatic breast cancer, with GEX data from *n* = 124 primary tumours and *n* = 74 distant metastases. This study population and acquisition of GEX data have been described previously [14, 15].

### Hormone receptor analyses and cut-offs

**Figure 2:**
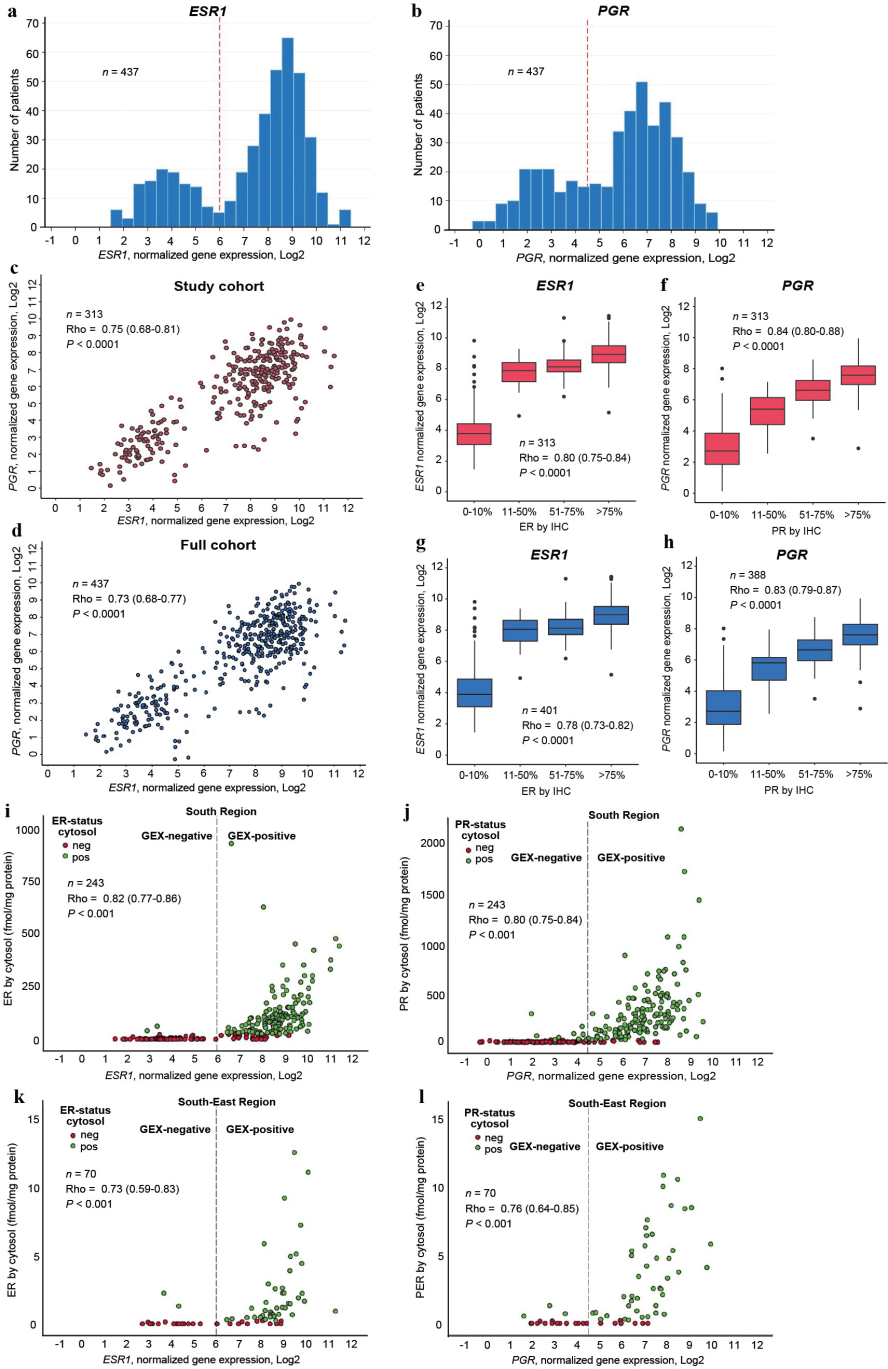
(A-L). Data distribution for all three ER and PR measuring methods. For all correlations presented, Rho is the Spearman’s correlation coefficient and p the p-value for a two-sided test of the null hypothesis Rho = 0. Distribution of mRNA expression levels of ESR1 (A) and PGR (B) including all patients with available GEX data. Red reference line at cut-off for positive ER and PR status by GEX. Relationship between mRNA expression levels of ESR1 and PGR in (C) all patients with data available from all three methods for ER and PR measurements, (D) all patients with ESR1 and PGR mRNA expression data available. Relationship between mRNA expression and IHC data for (E) ER and ESR1 in all patients with data available from all three methods for ER and PR measurements and (F) ER and ESR1 in all patients with IHC and mRNA data available, (G) PR and PGR in all patients with data available from all three methods for ER and PR measurements and (H) PR and PGR in all patients with IHC and mRNA data available. Relationship between mRNA expression and quantitative cytosol-based data for (I) ER and ESR1 in the South Health Care Region, (J) PR and PGR in the South Health Care Region, (K) ER and ESR1 in the South-East Health Care Region, (L) PR and PGR in the South-East Health Care Region. Cytosol: cytosol-based method; ER: Estrogen receptor; IHC: immunohistochemistry; GEX: gene expression; n: number of patients; PR: Progesterone receptor.

Areas of haematoxylin and eosin-stained tumour sections containing invasive carcinoma were marked and macro dissected as previously described [16]. Hormone receptor status was determined on fresh-frozen tumour tissue using cytosol-based methods as previously described [10, 12, 17, 18]. For ER determination, isoelectric focussing (IF) in polyacrylamide gels or enzyme-linked immunoassays (EIA) were used [10]. For PR determination, EIA, IF, or dextran-coated charcoal (DCC) with Scatchard analysis was used [10]. Cut-off for positive ER/PR status was ≥ 0.10 (IF) or ≥0.25 (EIA) fmol/ug DNA or ≥10 (IF and DCC) or ≥ 25 (EIA) fmol/mg protein according to guidelines at the two study centres at the time of the original study [10]. Immunohistochemistry analyses were performed on tissue microarrays from paraffin-embedded tumour samples (ER) or whole tissue sections from paraffin-embedded blocks collected in a follow-up study in 2020 (PR). Cut-off for positive ER/PR status by IHC was > 10% [10]. Antibodies and protocols for IHC and RNA extraction are described previously [10, 16]. mRNA expression was determined by Nanostring Technologies using the Nanostring Breast Cancer 360^TM^ assay on the Nanonstring nCounter© as previously described [16, 19]. GEX values are log-transformed relative mRNA measurements normalized to the expression of housekeeping genes. To identify biologically relevant cut-offs for positive ER and PR status based on GEX data, histograms demonstrating *ESR1* and *PGR* mRNA levels were depicted (Figure 2A–B) [20]. Cut-offs were selected using the visual appearance of the bimodal distributions of the two genes, independently of outcome. Positive GEX was defined as ≥ 6 (*ESR1)* and ≥ 4.5 (*PGR).*

### Follow-up and endpoints

Follow-up data from regional cancer centres, the Swedish Cause of Death Registry, and the Swedish Cancer Registry were collected as previously described [10, 11, 16]. Data cut-off was 20/12-2020 [16]. Endpoints were RFi and OS. Recurrence-free interval was defined as the time to a first event of recurrence (ipsilateral/local/regional/distant), ipsilateral ductal carcinoma *in situ* (DCIS), or death from breast cancer according to the Definition for the Assessment of Time-to-event Endpoints in Cancer trialist (DATECAN) guidelines for endpoints at the time of the original study [21]. OS was defined as the time from randomization to death from all causes.

### Ethics

The original study was approved by the ethical committees in Lund and Linköping. The follow-up study including information from patient records, the Swedish Cause of Death Registry, and the Swedish Cancer Registry was approved by the ethical committee of Lund University (LU 2015/350). Tissue retrieval for updated biomarker analyses and GEX analyses was approved by the ethical committee of Lund University (LU 2017/97).

### Statistical analyses

The agreement between the three methods was estimated using Fleiss’s *kappa*. Relations between continuous ER and PR data were assessed using Spearman’s Rho. Patients with available data from all three methods for both ER and PR were included in the survival analyses and treatment-interaction tests. Kaplan Meier graphs were used to demonstrate RFi and OS according to hormonal receptor status. Cox univariable regression was used to estimate hazard ratios (HR). Interaction between a dichotomous biomarker and tamoxifen treatment on the outcome was tested in Cox models including biomarker status, tamoxifen treatment, and an interaction term between the two dichotomous variables. HR_interaction_ is presented for the multiplicative interaction term between tamoxifen treatment and hormone receptor status. A HR_interaction_ = 1.00 reflects an effect of treatment which does not vary with hormone receptor status. A HR_interaction_≠1.00 suggests that positive hormone receptor status is associated with tamoxifen treatment being less effective (HR_interaction_ > 1.00) or more effective (HR_interaction_ < 1.00) in preventing the event of interest (recurrence or death).

All analyses were performed using the intention-to-treat rule. Nominal *p*-values presented should be cautiously interpreted as evidence against each null hypothesis without reference to a cut-off for significance. Results are presented adhering to the Recommendation of Tumor Marker Prognostic Studies (REMARK) criteria [22]. Statistical calculations were performed using IBM SPSS Statistics, version 28.0. Histograms and Kaplan Meier graphs were created using STATA, version 17.0. Boxplots were constructed in R 4.2.2. using RStudio 2023.06.0.

## Results

Patient and tumour characteristics are presented in Table 1. The two study arms within the study cohort with available hormone receptor status by all three methods (*n* = 313) were well balanced. For the 313 patients with hormone receptor status by all methods, median follow-up for event-free patients was 26 (RFi) and 33 (OS) years.

**Table 1:** Patient and tumour characteristics by study arm; for the whole study cohort, for the subgroup with available hormone receptor status analysed by all three methods, and for the excluded subgroup without available hormone receptor status analysed by all three methods.

Characteristics	Whole study cohort (*n* = 560)		Cohort with available hormone receptor status analysed by all three methods (*n* = 313)		Cohort without hormone receptor status analysed by all three methods (*n* = 247)	
	TAM (*n* = 276) No. (%)	Control (*n* = 284) No. (%)	TAM (*n* = 153) No. (%)	Control (*n* = 160) No. (%)	TAM (*n* = 123) No (%)	Control (*n* = 124) No (%)
**Age (years)**						
Median	45	45	44	45	45	46
Range	25–57	26–58	25–57	26–53	30–56	27–58
>40	51 (19)	59 (21)	28 (18)	36 (23)	23 (19)	23 (19)
40–49	178 (65)	183 (64)	109 (71)	110 (69)	69 (56)	73 (59)
50–59	47 (17)	42 (15)	16 (11)	14 (9)	31 (25)	28 (23)
Missing data	0	0	0	0	0	0
**Tumor size (mm)**						
Median	25	22	25	25	25	21
Range	5–75	2–50	8–75	2–50	5–60	4–50
≤20	86 (31)	121 (43)	46 (30)	59 (37)	40 (33)	62 (50)
>20	189 (69)	163 (57)	107 (70)	101 (63)	82 (67)	62 (50)
Missing data	1	0	0	0	1	0
**Number of positive nodes**						
Median	1	1	1	2	2	1
Range	0–21	0–22	0–20	0–20	0–21	0–22
Node-positive	192 (70)	208 (74)	105 (69)	121 (76)	87 (71)	87 (70)
Node-negative	83 (30)	75 (27)	48 (31)	38 (24)	35 (29)	37 (30)
Missing data	1	1	0	1	1	0
**NHG**						
1	27 (11)	32 (12)	13 (9)	13 (8)	14 (14)	19 (18)
2	105 (42)	115 (44)	56 (38)	68 (43)	49 (49)	47 (45)
3	117 (47)	116 (44)	79 (53)	78 (49)	38 (38)	38 (37)
Missing data	27	21	5	1	22	20
**ER, IHC**						
Positive (>10%)	151 (66)	173 (71)	102 (67)	111 (69)	49 (65)	62 (74)
Negative (≤10%)	78 (34)	71 (29)	51 (33)	49 (31)	27 (36)	22 (26)
Missing data	47	40	0	0	47	40
**ER, cytosol**						
Positive	133 (60)	140 (61)	92 (60)	96 (60)	41 (59)	44 (62)
Negative	89 (40)	91 (39)	61 (40)	64 (40)	28 (41)	27 (38)
Missing data	54	53	0	0	54	53
**ER, GEX^a^**						
Positive	156 (72)	164 (75)	108 (71)	115 (72)	48 (75)	49 (82)
Negative	61 (28)	56 (25)	45 (29)	45 (28)	16 (25)	11 (18)
Missing data	59	64	0	0	59	64
**PR, IHC**						
Positive (>10%)	141 (62)	161 (69)	90 (59)	103 (64)	51 (67)	58 (77)
Negative (≤10%)	88 (38)	74 (32)	63 (41)	57 (36)	25 (33)	17 (23)
Missing data	47	49	0	0	47	49
**PR, cytosol**						
Positive	138 (63)	151 (65)	95 (62)	107 (67)	43 (65)	44 (62)
Negative	81 (37)	80 (35)	58 (38)	53 (33)	23 (35)	27 (38)
Missing data	57	53	0	0	57	53
**PR, GEX^b^**						
Positive	151 (70)	157 (71)	100 (65)	109 (68)	51 (80)	48 (80)
Negative	66 (30)	63 (29)	53 (35)	51 (32)	13 (20)	12 (20)
Missing data	59	64	0	0	59	64
**Ki67 (%)**						
Low 0–13	34 (15)	26 (11)	20 (13)	10 (6)	14 (19)	16 (21)
Intermediate 14–19	27 (12)	25 (11)	11 (7)	13 (8)	16 (21)	12 (16)
High 20–100	167 (73)	184 (78)	122 (80)	136 (86)	45 (60)	48 (63)
Missing data	48	49	0	1	48	48
**Subtype, St Gallen 2013**						
Luminal-like	132 (61)	148 (64)	85 (60)	93 (60)	47 (63)	55 (71)
HER2-positive	30 (14)	38 (16)	18 (13)	32 (21)	12 (16)	6 (8)
TNBC	54 (25)	46 (20)	38 (27)	29 (19))	16 (21)	17 (22)
Missing data	60	52	12	6	48	46
**PAM50**						
Luminal A	90 (42)	101 (46)	57 (37))	68 (43)	33 (52)	33 (55)
Luminal B	42 (19)	41 (19)	32 (21)	29 (18)	10 (16)	12 (20)
HER2-E	35 (16)	39 (18)	24 (16)	36 (23)	11 (17)	3 (5)
Basal	50 (23)	39 (18)	40 (26)	27 (17)	10 (16)	12 (20)
Missing data	59	64	0	0	59	64

GEX: gene expression; TAM: tamoxifen; NHG: Nottingham histological grade; ER: estrogen receptor; IHC: immunohistochemistry; PR: progesterone receptor; HER2: human epidermal growth factor receptor 2; TNBC: triple-negative breast cancer; PAM50: prediction analysis of microarray 50; HER2-E: human epidermal growth factor receptor 2-enriched.

a Positive ER-status by mRNA expression of *ESR1* defined as ≥ 6 on the normalised logarithmic scale presented from Nanostring.

b Positive PR-status by mRNA expression of *PGR* defined as ≥ 4.5 on the normalised logarithmic scale presented from Nanostring.

### ESR1 and PGR GEX and its correlation to protein expression

mRNA levels of *ESR1* and *PGR* within the study population appeared to be bimodally distributed (Figure 2A–B). To evaluate whether this data-driven cutoff may be generalisable to other cohorts and thus a feasible clinical strategy, we assessed the distribution of *ESR1* and *PGR* expression in an independent cohort of metastatic breast cancer that includes GEX data from primary tumours and distant metastases. Indeed, the *ESR1* and *PGR* GEX in that cohort exhibited bimodal distributions comparable to the patterns in the current study (Supplementary Figure S1). Additionally, for both genes, the distribution of the primary tumours and the distant metastases were similar, with consistent cutoffs.

The correlation between *ESR1* and *PGR* mRNA was 0.75 (Rho, 95% confidence interval [CI] 0.68–0.81, *p* < 0.0001) (Figure 2C). The relationship was similar when all samples with available mRNA data were included (Figure 2D). A strong correlation between mRNA expression and protein expression by IHC was found for ER (Rho 0.80, 95% CI 0.75–0.84, *p* < 0.0001) (Figure 2E) as well as for PR (Rho 0.84, 95% CI 0.80–0.88, *p* < 0.0001) (Figure 2F). When all samples with available mRNA data were included, corresponding patterns were observed (Figure 2G–Hh). Additionally, mRNA expression correlated with protein expression by cytosol-based methods from both study centres, for ER (Figure 2I–K) and PR (Figure 2J–L).

### Concordance between the three methods

Number of patients for each combination of positive and negative results for the three methods is demonstrated in Supplementary Table S1. Between all three methods, concordance for ER was 84% (*kappa* 0.76, 95% CI 0.69–0.82, *p* < 0.0001) and for PR 85% (*kappa* 0.79, 95% CI 0.72–0.85, *p* < 0.0001). Because of the small number of patients with discordant ER and PR status: 51 (16%) and 46 (15%) respectively, no survival statistical analysis including only patients with discordant results was performed. The number of patients that were hormone receptor-positive by GEX but negative by IHC was 12 (ER) and 19 (PR). The opposite discordant pattern, that is, hormone receptor-negative by GEX but positive by IHC was observed for two (ER) and three (PR) patients, respectively.

### Tamoxifen effect in terms of RFi and OS

**Figure 3:**
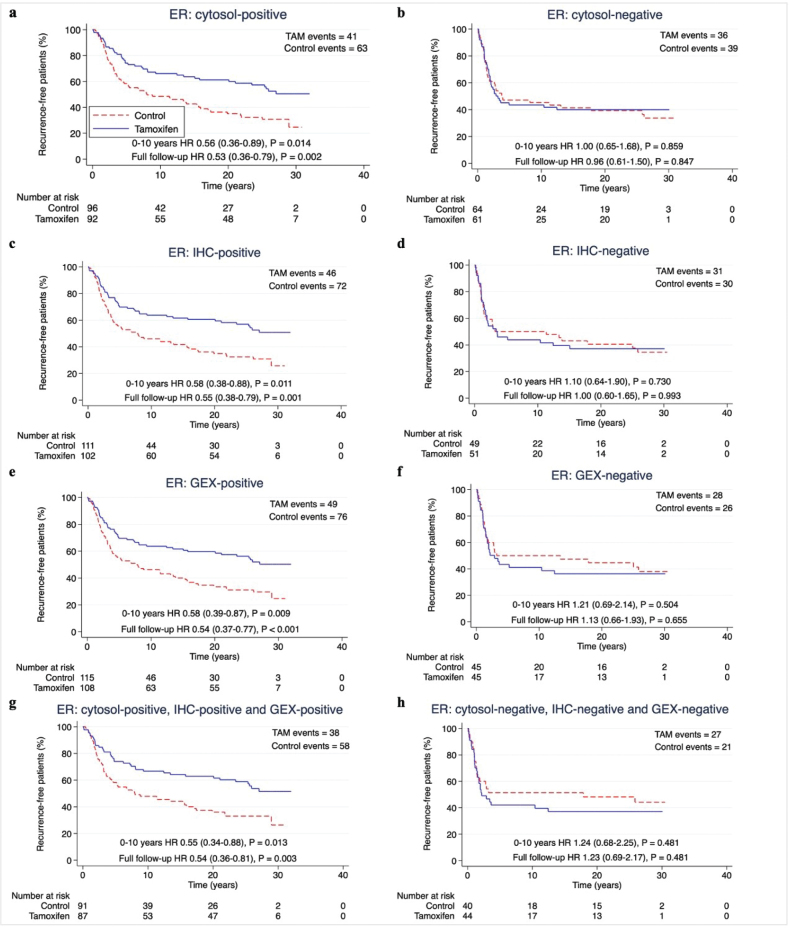
(A–H) Recurrence-free interval according to positive or negative ER status by different methods. Patients with available data for all methods for ER and PR were included (n = 313). P-value by log-rank test, 5% significance level. (A–B) cytosol-based methods. (C–D) IHC, (E–F) gene expression, (G-H) hormone receptor status for all methods. ER: Estrogen receptor; PR: Progesterone receptor; cytosol: cytosol-based method; IHC: immunohistochemistry; GEX: gene expression; TAM: Tamoxifen.

**Figure 4:**
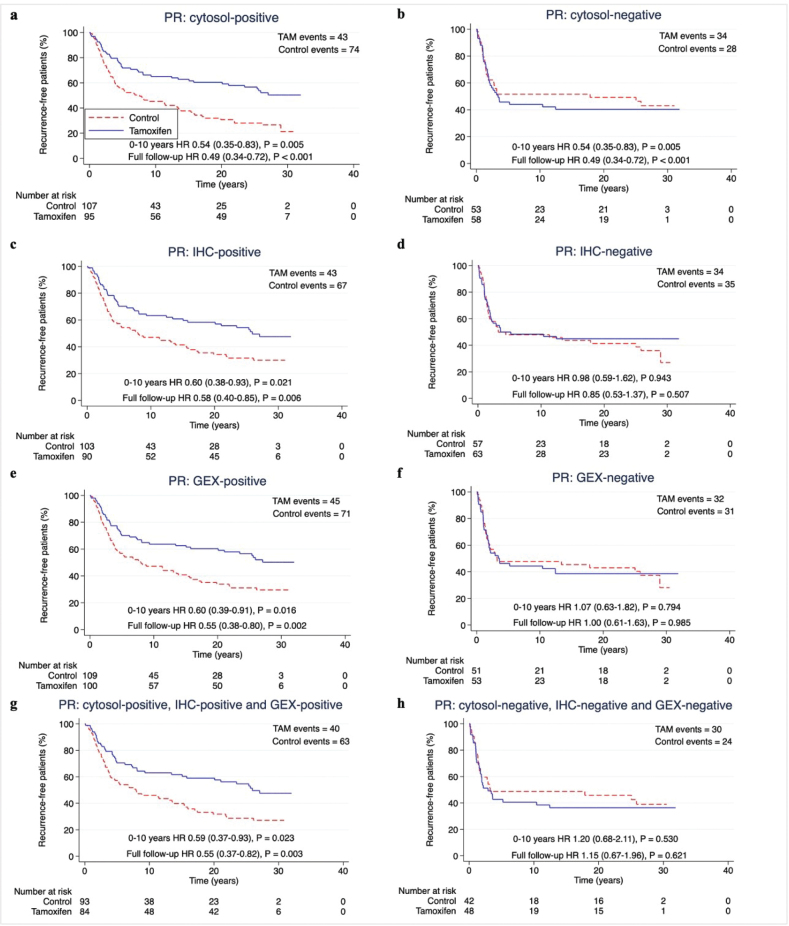
(A–H) Recurrence-free interval according to positive or negative PR status by different methods. Patients with available data for all methods for ER and PR were included (n = 313). P-value by log-rank test, 5% significance level. (A–B) cytosol-based methods. (C–D) IHC, (E–F) gene expression, (G–H) hormone receptor status for all methods. PR: Progesterone receptor; ER: Estrogen receptor; cytosol: cytosol-based method; IHC: immunohistochemistry; GEX: gene expression; TAM: Tamoxifen.

To determine the predictive performance of each hormone receptor measuring method, we assessed the benefit of tamoxifen in subgroups classified as receptor-positive according to each method separately. Two years of tamoxifen prolonged RFi at 10 years and at full follow-up in patients with ER-positive (ER+) or PR-positive (PR+) tumours by each method (HR and 95% CI) at 10 years: cytosol-ER+ 0.56 (0.36–0.89), *p =* 0.014; IHC-ER+ 0.58 (0.38–0.88), *p* = 0.011; GEX-ER+ 0.58 (0.39–0.87), *p* = 0.009; cytosol-PR+ 0.54 (0.35–0.83), *p* = 0.005; IHC-PR+ 0.60 (0.38–0.93), *p* = 0.021; GEX-PR+ 0.60 (0.39–0.91), *p* = 0.016. HRs at full follow-up time: cytosol-ER+ 0.53 (0.36–0.79), *p* = 0.002; IHC-ER+ 0.55 (0.38–0.79), *p* = 0.001; GEX-ER+ 0.54 (0.37–0.77), *p* < 0.001; cytosol-PR+ 0.49 (0.34–0.72), *p* < 0.001; IHC-PR+ 0.58 (0.40–0.85), *p* = 0.006; GEX-PR+ 0.55 (0.38–0.80), *p* = 0.002) (Figures 3–4 and Table 2).

**Table 2:** Cox univariate regression (n = 313).

	10-year RFi		10-year OS		Full follow-up time RFi		Full follow-up time OS	
	HR (95% CI)	*p*	HR (95% CI)	*p*	HR (95% CI)	*p*	HR (95% CI)	*p*
**ER**								
Cytosol-positive (*n* = 188)	0.56 (0.36–0.89)	0.014	0.75 (0.46–1.24)	0.265	0.53 (0.36–0.79)	0.002	0.62 (0.44–0.89)	0.010
Cytosol-negative (*n* = 125)	1.00 (0.65–1.68)	0.859	1.02 (0.63–1.65)	0.947	0.96 (0.61–1.50)	0.847	0.90 (0.60–1.35)	0.615
IHC-positive (*n* = 213)	0.58 (0.38–0.88)	0.011	0.83 (0.53–1.30)	0.407	0.55 (0.38–0.79)	0.001	0.65 (0.47–0.91)	0.013
IHC-negative (*n* = 100)	1.10 (0.64–1.90)	0.730	0.91 (0.53–1.56)	0.728	1.00 (0.60–1.65)	0.993	0.88 (0.55–1.38)	0.568
GEX-positive (*n* = 223)	0.58 (0.39–0.87)	0.009	0.79 (0.51–1.23)	0.287	0.54 (0.37–0.77)	<0.001	0.65 (0.47–0.90)	0.010
GEX-negative (*n* = 90)	1.21 (0.69–2.14)	0.504	1.03 (0.59–1.80)	0.926	1.13 (0.66–1.93)	0.655	0.93 (0.57–1.51)	0.757
Triple-positive (*n* = 178)	0.55 (0.34–0.88)	0.013	0.78 (0.47–1.30)	0.337	0.54 (0.36–0.81)	0.003	0.63 (0.44–0.92)	0.016
Triple-negative (*n* = 84)	1.24 (0.68–2.25)	0.481	1.02 (0.57–1.84)	0.938	1.23 (0.69–2.17)	0.481	0.97 (0.58–1.62)	0.911
**PR**								
Cytosol-positive (*n* = 202)	0.54 (0.35–0.83)	0.005	0.77 (0.48–1.24)	0.290	0.49 (0.34–0.72)	<0.001	0.60 (0.42–0.84)	0.003
Cytosol-negative (*n* = 111)	1.17 (0.69–1.97)	0.568	0.95 (0.57–1.59)	0.848	1.12 (0.68–1.85)	0.657	0.96 (0.62–1.50)	0.853
IHC-positive (*n* = 193)	0.60 (0.38–0.93)	0.021	0.86 (0.53–1.39)	0.540	0.58 (0.40–0.85)	0.006	0.69 (0.48–0.97)	0.035
IHC-negative (*n* = 120)	0.98 (0.59–1.62)	0.943	0.84 (0.51–1.38)	0.483	0.85 (0.53–1.37)	0.507	0.78 (0.51–1.18)	0.776
GEX-positive (*n* = 209)	0.60 (0.39–0.91)	0.016	0.87 (0.55–1.38)	0.541	0.55 (0.38–0.80)	0.002	0.64 (0.45–0.89)	0.009
GEX-negative (*n* = 104)	1.07 (0.63–1.82)	0.794	0.86 (0.51–1.45)	0.574	1.00 (0.61–1.63)	0.985	0.91 (0.58–1.43)	0.690
Triple-positive (*n* = 177)	0.59 (0.37–0.93)	0.023	0.91 (0.55–1.52)	0.727	0.55 (0.37–0.82)	0.003	0.67 (0.47–0.97)	0.035
Triple-negative (*n* = 90)	1.20 (0.68–2.11)	0.530	0.98 (0.56–1.73)	0.955	1.15 (0.67–1.96)	0.621	1.01 (0.62–1.65)	0.954

ER: estrogen receptor; PR: progesterone-receptor; RFi: recurrence-free interval; OS: overall survival; HR: hazard ratio; CI: confidence interval; cytosol: cytosol-based method; IHC: immunohistochemistry; GEX: gene expression.

The predictive performance of the three hormone receptor measuring methods was equal also with regards to OS, with HRs for tamoxifen treatment at 10 years follow-up around 0.80 for patients with tumours ER+ or PR+ by any method, and around 0.65 at full follow-up (Supplementary Figure S2–S3 and Table 2).

When assessing the effect of tamoxifen on ‘triple-positive’ tumours (positive by all available methods), the HR for RFi and OS for tamoxifen was equal to each measuring method separately for both ER and PR (Table 2).

### Interaction analysis

Interaction effects on RFi at full-follow up time between tamoxifen treatment and ER or PR status was on average almost a factor two downwards, that is a two-fold larger reduction of incidence of events in receptor-positive compared to receptor-negative patients (HR_interaction_ (95% CI),ER-cytosol 0.60 (0.33–1.09), *p* = 0.095; ER-IHC 0.56 (0.30–1.04), *p* = 0.067; ER-GEX 0.48 (0.25–0.92), *p* = 0.026; PR-cytosol 0.46 (0.24–0.85), *p* = 0.014; PR-IHC 0.73 (0.40–1.35), *p* = 0.32; PR-GEX 0.58 (0.31–1.08), *p* = 0.084) as demonstrated in Table 3. Corresponding figures for interactions at 10 years’ follow-up are displayed in Table 3. The evidence against the null hypothesis that the effect of tamoxifen was equal regardless of ER or PR status, was lower in the analysis of interactions on OS compared to RFi (Table 3).

**Table 3:** Interaction HR for the interaction effect between ER or PR status by each method and tamoxifen treatment (n = 313).

	10-year RFI		10-years OS		Full follow-up RFi		Full follow-up OS	
	HR (95% CI)	*p*	HR (95% CI)	*p*	HR (95% CI)	*p*	HR (95% CI)	*p*
ER, cytosol	0.56 (0.29–1.09)	0.087	0.73 (0.36–1.46)	0.371	0.60 (0.33–1.09)	0.095	0.73 (0.43–1.26)	0.257
ER, IHC	0.54 (0.27–1.06)	0.074	0.91 (0.45–1.84)	0.785	0.56 (0.30–1.04)	0.067	0.79 (0.45–1.38)	0.400
ER, GEX	0.48 (0.24–0.97)	0.041	0.75 (0.37–1.53)	0.429	0.48 (0.25–0.92)	0.026	0.74 (0.41–1.32)	0.302
PR, cytosol	0.47 (0.24–0.94)	0.031	0.81 (0.40–1.63)	0.533	0.46 (0.24–0.85)	0.014	0.66 (0.38–1.15)	0.142
PR, IHC	0.64 (0.33–1.25)	0.192	1.03 (0.52–2.07)	0.931	0.73 (0.40–1.35)	0.315	0.95 (0.55–1.64)	0.855
PR, GEX	0.58 (0.30–1.14)	0.116	1.01 (0.50–2.03)	0.977	0.58 (0.31–1.08)	0.084	0.73 (0.42–1.28)	0.274

HR_interaction_ are presented for the multiplicative interaction term between hormone receptor status and tamoxifen treatment.

RFi: recurrence-free interval; OS: overall survival; HR: hazard ratio; CI: confidence interval; ER: estrogen receptor; PR: progesterone receptor; IHC: immunohistochemistry; GEX: gene expression.

## Discussion

We demonstrate that positive ER or PR status measured by cytosol-based methods, IHC, and GEX analysis, is predictive of tamoxifen benefit for premenopausal patients with invasive breast cancer. The methods were equally good at predicting benefit of tamoxifen supporting that the methods can be interchangeable. Tamoxifen is still widely used as an adjuvant therapy in premenopausal women and the present finding that mRNA levels of hormone receptors carry tamoxifen predictive information is thus clinically relevant. In the present trial, 2 years of tamoxifen was evaluated in contrast to today’s recommendation of five or more years. However, prediction of the effect of 2 years of tamoxifen is today important for patients aiming to get pregnant after a breast cancer diagnosis, since these patients will be recommended to interrupt adjuvant endocrine therapy after 2 years. Additionally, side effects prevent some patients from completing the specified treatment course of at least 5 years [23].

mRNA levels of *ESR1* and *PGR* had bimodal distributions, suggesting there may be a clear discrimination between patients with hormone receptor-positive and receptor -negative tumours at the mRNA level. To our knowledge, no previous publication has demonstrated this bimodal distribution of mRNA levels of *ESR1* and *PGR*. Furthermore, mRNA levels strongly correlated to protein expression analysed by cytosol-based methods and IHC, indicating that mRNA levels are a good surrogate marker for protein expression when analysing hormone receptors.

In line with our findings, where concordances between all three methods were 84% (ER) and 85% (PR), previous studies have reported concordance around 87% (ER) and 86% (PR) between cytosol-based methods and IHC for assessment of protein expression, and around 92% (ER) and 89% (PR) between protein expression by IHC and GEX analysis [24–27]. The slightly lower concordance reported in the present study may be due to the comparison of three methods instead of two, and the relatively small number of exclusively premenopausal patients included. To our knowledge, no previous publication has compared all three methods.

Patients receiving tamoxifen had longer RFi if their tumour was ER+ or PR+ regardless of the applied method for receptor determination. In patients with tumours ER+ or PR+ by either method, HRs for tamoxifen were similar, indicating that the methods are equally good at identifying patients who benefit from tamoxifen. Combining all three methods to consider ‘triple-positive’ tumours did not seem to improve the predictive performance, as ‘triple-positive’ tumours had equal benefit of tamoxifen as ‘single-positives’. These results suggest that cytosol-based methods or IHC assessment for protein expression, and GEX analysis of hormone receptors perform equally well and are interchangeable as predictive tools. A larger cohort study would be needed to determine this. A larger study could also address patients with discordant results that in this study, due to the small number (ER 51/313; PR 46/313), were not further tested statistically. Twelve patients were GEX-ER+ but IHC-ER-, and two patients were IHC-ER+ but GEX-ER-. While our data suggests that hormone receptor status by GEX is equal to IHC as a predictive tool, it is unclear how to interpret the discordant results. To evaluate the tamoxifen benefit of patients identified as ER-positive by GEX but not by IHC and vice versa, a larger study including more patients with discordant results between the two methods is needed.

It is well known that cytosol-based methods and IHC for determining hormone receptor status can be used for prediction of tamoxifen benefit [3, 9]. Interestingly, this study found that GEX of *ESR1* and *PGR* could be used as well. In a study by Kim et al. [5] patients were randomized to tamoxifen or placebo irrespectively of menopausal status and distant RFi at 10 years were compared according to mRNA levels of *ESR1* and *PGR*. The authors found that high *ESR1* mRNA levels were associated with tamoxifen benefit, while GEX of *PGR* lacks predictive function [5]. Throughout this study, also positive PR status determined by any method seemed to be predictive of tamoxifen benefit. This result may differ from the EBCTCG report that stated that, given the ER status, PR status does not give any additional predictive information [3]. In the present study, including premenopausal patients only, most PR+ patients were ER+, which might be a reason for the tamoxifen response observed in the PR+ subgroups. Nevertheless, several studies have demonstrated a predictive value for PR independently of ER status [28–30].

In the analysis of OS at 10 years, evidence for tamoxifen benefit was lower compared to the analysis of RFi. Although, HRs for patients positive for ER and PR by any method were lower in the tamoxifen arm than in the control arm. With around three decades of follow-up, tamoxifen improved OS, as previously reported by Ekholm et al. [11] for patients with ER+ tumours in this trial. They also found a trend of decreased cumulative mortality (including death of all causes) for ER+ patients receiving tamoxifen for the same follow-up time. In a study by Khoshnoud et al. [24], IHC, and cytosol-based methods for determination of ER status in postmenopausal women were compared regarding the ability to predict benefit of tamoxifen. Patients were randomised to 2 years of adjuvant tamoxifen or no systemic treatment [24]. The authors found that both IHC and cytosol-based methods could identify patients that had benefit from tamoxifen in terms of recurrence-free survival, but also had lower evidence against the null hypothesis of no difference in OS. The authors concluded that IHC and cytosol-based methods were interchangeable for predicting tamoxifen benefit, which supports the results of the present study [24].

For RFi, ER status by GEX analysis and PR-status by cytosol-based methods had the lowest interaction *p*-values with tamoxifen treatment. In the study by Kim et al. [5], the authors also found a tamoxifen treatment interaction with high mRNA levels of *ESR1* in relation to distant RFi. A larger study population is needed for more statistical power and reliability in interaction analyses.

Strengths of this study are its randomised design, long follow-up, and that most of the patients only received tamoxifen as systemic treatment, or no systemic treatment. The latter provides an opportunity to study the effects of tamoxifen independent of other systemic treatments. Another strength is that we provide a proof-of-concept analysis for the proposed data-driven cutoff strategy for GEX of *ESR1* and *PGR* in an independent cohort of metastatic breast cancer. Observing that GEX of both genes is bimodally distributed in both primary tumours and distant metastases – with similar cutoff – suggests that this method is transferrable to other cohorts and tumour stages.

Limitations include the number of patients. Selecting 313 patients, with data available for all three measuring methods, out of 564 patients originally randomised could lead to bias. However, patients and tumour characteristics of the included and excluded subgroups showed no noticeable differences between the two groups (Table 1). A limitation in transferability includes the use of the cut-off > 10% for IHC, which differs from the cut-offs often used globally [4]. Furthermore, the cytosol-based hormone determinations were conducted at the time of the original surgery, while IHC and GEX analyses were done retrospectively. Since protein and RNA can degrade over time, the long timespan between analyses could affect the results.

Today patients are given at least 5 years of tamoxifen [9, 31]. However, 5 to 10 years of treatment can be problematic for premenopausal patients wishing to become pregnant, making studies of shorter treatment regimens of tamoxifen interesting. Importantly, the recently presented trial by Partridge et al. [32] concluded that interruption of endocrine treatment after 18–30 months, was not associated with an increased risk of recurrence compared to an external control cohort which did not interrupt treatment. Furthermore, when the SBII:2pre trial was conducted, the overall prognosis for premenopausal breast cancer patients was worse than today, that is HER2-positive patients included did not receive trastuzumab, which would have affected their prognosis [33].

The finding that GEX of *ESR1* and *PGR* can be used to predict tamoxifen benefit is clinically useful since GEX analysis is already routinely performed in patient subgroups with early breast cancer. Importantly, several gene assays used for this purpose, including Oncotype DX^®^ and Prosigna^®^, include data on *ESR1* and *PGR* expression [7–9, 19, 34]. In the future, *ESR1* and *PGR* data from these patients could potentially be used for hormone receptor determination as an alternative to IHC.

## Conclusion

In the present study, three methods for determination of hormone receptor status were applied in relation to RFi and OS in premenopausal breast cancer patients treated with tamoxifen in comparison to a control group in a randomised trial with long-term follow-up. Breast tumour measurement of hormone receptors by protein expression through cytosol-based methods and IHC, as well as mRNA levels by GEX analysis, can all identify patients that benefit from 2 years of tamoxifen. The methods seem equally good at predicting tamoxifen benefit, indicating that also mRNA data might be used to guide adjuvant tamoxifen therapy.

## Supplementary Material

Hormone receptor mRNA and protein levels as predictors of premenopausal tamoxifen benefit

Hormone receptor mRNA and protein levels as predictors of premenopausal tamoxifen benefit

## Data Availability

The data supporting the findings of this study are not publicly available due to restrictions related to data sensitivity, but available from the corresponding author upon reasonable request.
