# Supplementary material for: Hormone receptor mRNA and protein levels as predictors of premenopausal tamoxifen benefit
Source: Acta Oncol. 2024 Apr 8;63:19655. doi: 10.2340/1651-226X.2024.19655 (PMC11332536; doi:10.2340/1651-226X.2024.19655)
Supplement: Hormone receptor mRNA and protein levels as predictors of premenopausal tamoxifen benefit [file AO-63-19655-s1.pdf]

Supplementary material has been published as submitted. It has not been copyedited or typeset by Acta Oncologica.

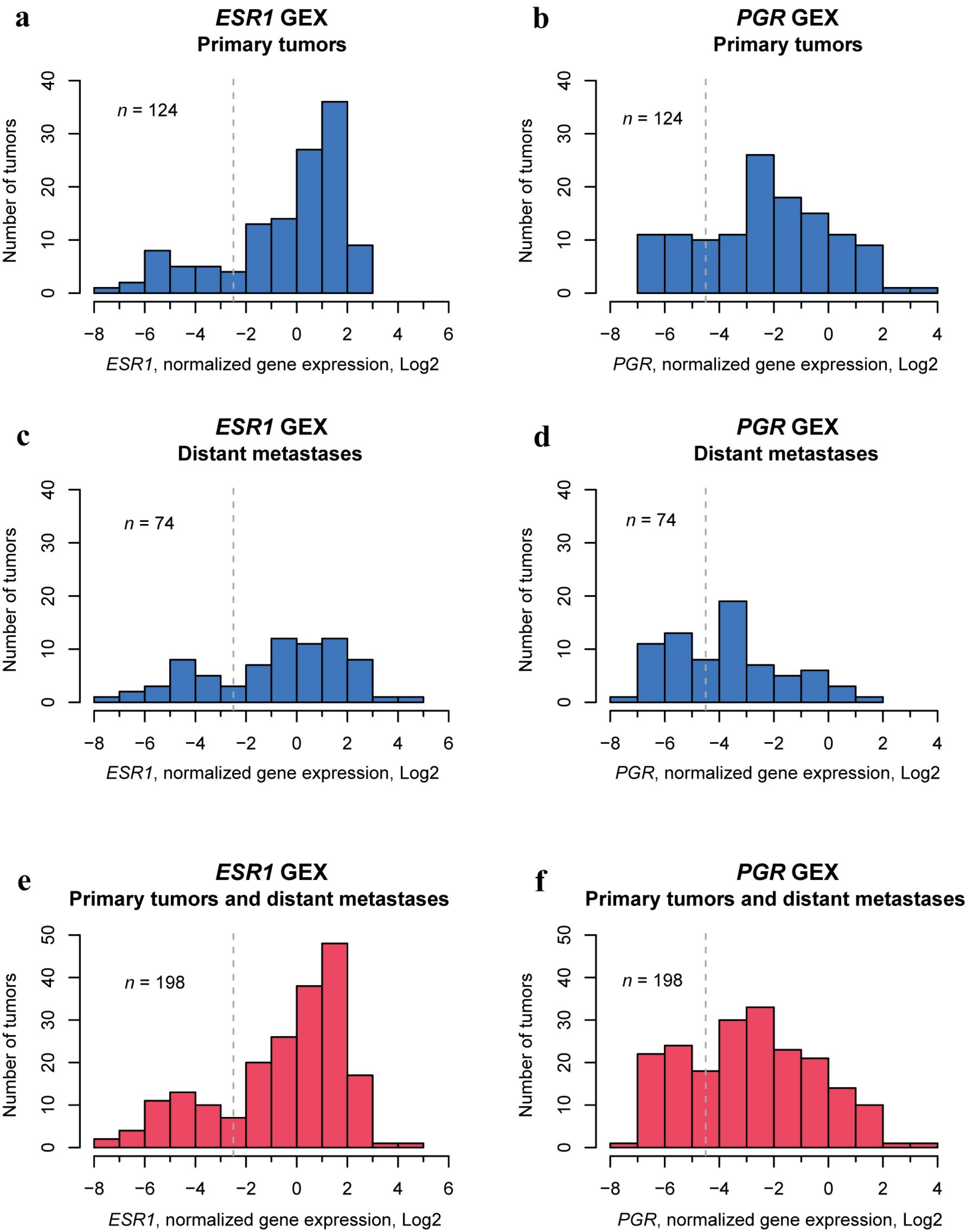
a
ESR1 GEX
Primary tumors
n = 124
Number of tumors
ESR1, normalized gene expression, Log2
b
PGR GEX
Primary tumors
n = 124
Number of tumors
PGR, normalized gene expression, Log2
c
ESR1 GEX
Distant metastases
n = 74
Number of tumors
ESR1, normalized gene expression, Log2
d
PGR GEX
Distant metastases
n = 74
Number of tumors
PGR, normalized gene expression, Log2
e
ESR1 GEX
Primary tumors and distant metastases
n = 198
Number of tumors
ESR1, normalized gene expression, Log2
f
PGR GEX
Primary tumors and distant metastases
n = 198
Number of tumors
PGR, normalized gene expression, Log2

Figure S1

Distribution of mRNA expression levels of ESR1 (a, c, e) and PGR (b, d, f) in an independent cohort of patients with metastatic breast cancer containing GEX data from paired primary tumor and distant metastasis samples previously described in detail (14, 15). The data distribution is shown separately for primary tumors (a-b), distant metastasis (c-d), and both primary tumors and distant metastases pooled together (e-f). The vertical grey dotted lines mark a suggested cutoff for positive/negative ESR1 and PGR status respectively, selected as described in the manuscript.

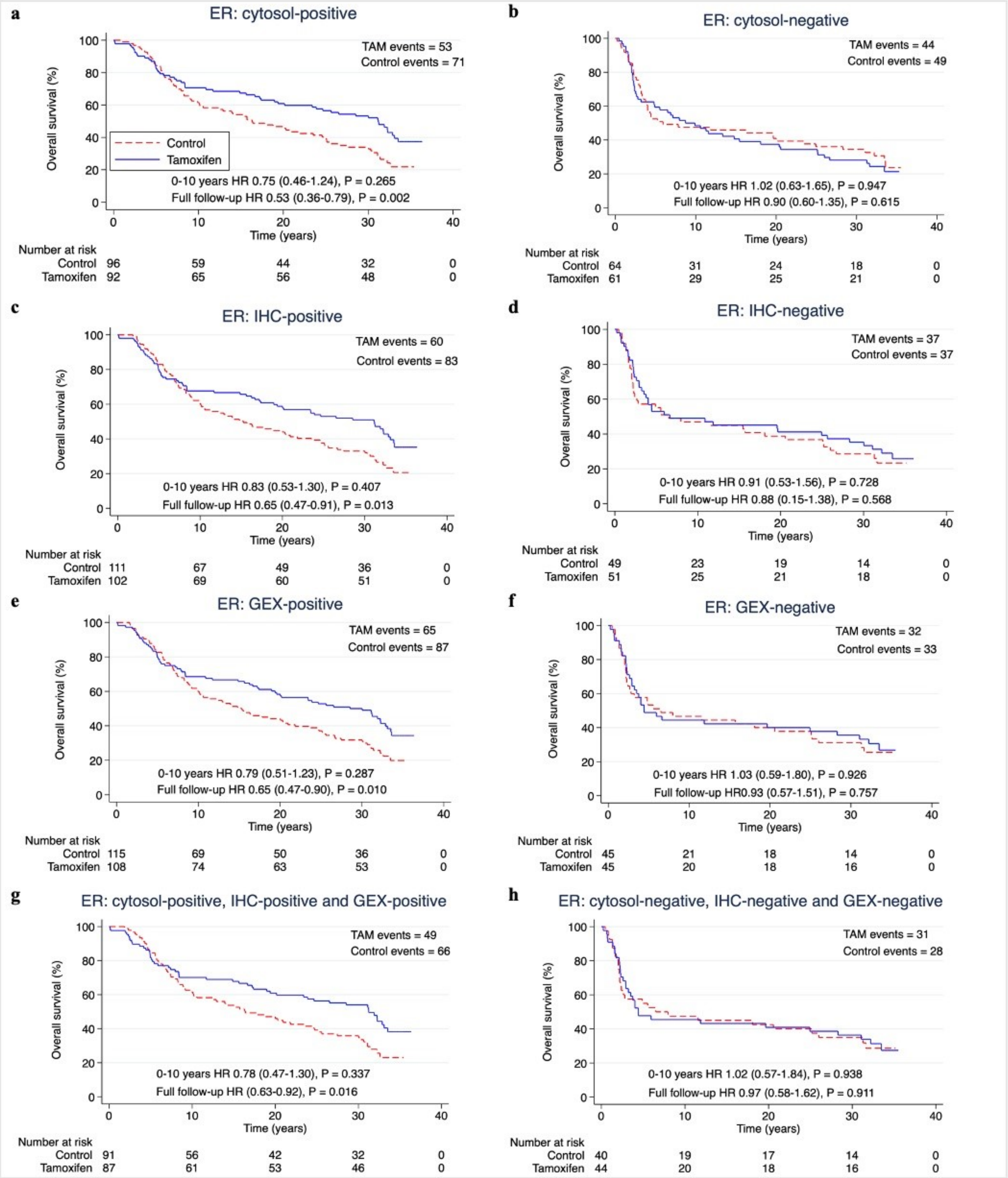
a
ER: cytosol-positive
TAM events = 53
Control events = 71
Control
Tamoxifen
0-10 years HR 0.75 (0.46-1.24), P = 0.265
Full follow-up HR 0.53 (0.36-0.79), P = 0.002
Overall survival (%)
Time (years)
Number at risk
Control 96 59 44 32 0
Tamoxifen 92 65 56 48 0
b
ER: cytosol-negative
TAM events = 44
Control events = 49
0-10 years HR 1.02 (0.63-1.65), P = 0.947
Full follow-up HR 0.90 (0.60-1.35), P = 0.615
Overall survival (%)
Time (years)
Number at risk
Control 64 31 24 18 0
Tamoxifen 61 29 25 21 0
c
ER: IHC-positive
TAM events = 60
Control events = 83
0-10 years HR 0.83 (0.53-1.30), P = 0.407
Full follow-up HR 0.65 (0.47-0.91), P = 0.013
Overall survival (%)
Time (years)
Number at risk
Control 111 67 49 36 0
Tamoxifen 102 69 60 51 0
d
ER: IHC-negative
TAM events = 37
Control events = 37
0-10 years HR 0.91 (0.53-1.56), P = 0.728
Full fullow-up HR 0.88 (0.15-1.38), P = 0.568
Overall survival (%)
Time (years)
Number at risk
Control 49 23 19 14 0
Tamoxifen 51 25 21 18 0
e
ER: GEX-positive
TAM events = 65
Control events = 87
0-10 years HR 0.79 (0.51-1.23), P = 0.287
Full follow-up HR 0.65 (0.47-0.90), P = 0.010
Overall survival (%)
Time (years)
Number at risk
Control 115 69 50 36 0
Tamoxifen 108 74 63 53 0
f
ER: GEX-negative
TAM events = 32
Control events = 33
0-10 years HR 1.03 (0.59-1.80), P = 0.926
Full follow-up HR0.93 (0.57-1.51), P = 0.757
Overall survival (%)
Time (years)
Number at risk
Control 45 21 18 14 0
Tamoxifen 45 20 18 16 0
g
ER: cytosol-positive, IHC-positive and GEX-positive
TAM events = 49
Control events = 66
0-10 years HR 0.78 (0.47-1.30), P = 0.337
Full follow-up HR (0.63-0.92), P = 0.016
Overall survival (%)
Time (years)
Number at risk
Control 91 56 42 32 0
Tamoxifen 87 61 53 46 0
h
ER: cytosol-negative, IHC-negative and GEX-negative
TAM events = 31
Control events = 28
0-10 years HR 1.02 (0.57-1.84), P = 0.938
Full follow-up HR 0.97 (0.58-1.62), P = 0.911
Overall survival (%)
Time (years)
Number at risk
Control 40 19 17 14 0
Tamoxifen 44 20 18 16 0

Figure S2

Overall survival according to positive or negative ER status by different methods. Patients with available data for all methods for ER and PR were included (n=313). P-value by log-rank test, 5% significance level. a-b) cytosol-based method. c-d) IHC, e-f) gene expression, g-h) same result for all methods.

ER, Estrogen receptor; cytosol, cytosol-based method; IHC, immunohistochemistry; GEX, gene expression; PR, Progesterone receptor; TAM, Tamoxifen.

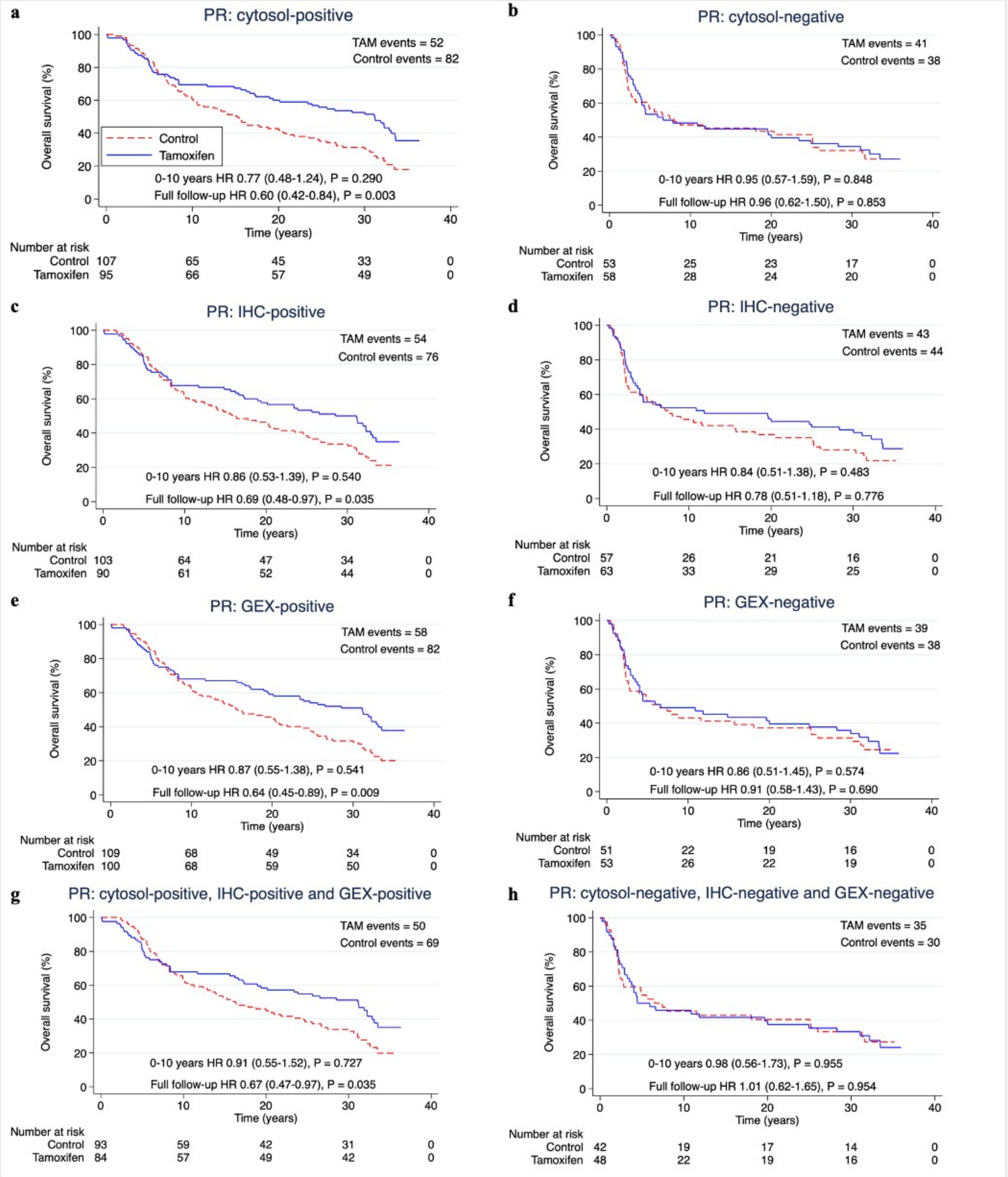

a
PR: cytosol-positive
TAM events = 52
Control events = 82
Control
Tamoxifen
0-10 years HR 0.77 (0.48-1.24), P = 0.290
Full follow-up HR 0.60 (0.42-0.84), P = 0.003
Overall survival (%)
Time (years)
Number at risk
Control 107 65 45 33 0
Tamoxifen 95 66 57 49 0
b
PR: cytosol-negative
TAM events = 41
Control events = 38
0-10 years HR 0.95 (0.57-1.59), P = 0.848
Full follow-up HR 0.96 (0.62-1.50), P = 0.853
Number at risk
Control 53 25 23 17 0
Tamoxifen 58 28 24 20 0
c
PR: IHC-positive
TAM events = 54
Control events = 76
0-10 years HR 0.86 (0.53-1.39), P = 0.540
Full follow-up HR 0.69 (0.48-0.97), P = 0.035
Number at risk
Control 103 64 47 34 0
Tamoxifen 90 61 52 44 0
d
PR: IHC-negative
TAM events = 43
Control events = 44
0-10 years HR 0.84 (0.51-1.38), P = 0.483
Full follow-up HR 0.78 (0.51-1.18), P = 0.776
Number at risk
Control 57 26 21 16 0
Tamoxifen 63 33 29 25 0
e
PR: GEX-positive
TAM events = 58
Control events = 82
0-10 years HR 0.87 (0.55-1.38), P = 0.541
Full follow-up HR 0.64 (0.45-0.89), P = 0.009
Number at risk
Control 109 68 49 34 0
Tamoxifen 100 68 59 50 0
f
PR: GEX-negative
TAM events = 39
Control events = 38
0-10 years HR 0.86 (0.51-1.45), P = 0.574
Full follow-up HR 0.91 (0.58-1.43), P = 0.690
Number at risk
Control 51 22 19 16 0
Tamoxifen 53 26 22 19 0
g
PR: cytosol-positive, IHC-positive and GEX-positive
TAM events = 50
Control events = 69
0-10 years HR 0.91 (0.55-1.52), P = 0.727
Full follow-up HR 0.67 (0.47-0.97), P = 0.035
Number at risk
Control 93 59 42 31 0
Tamoxifen 84 57 49 42 0
h
PR: cytosol-negative, IHC-negative and GEX-negative
TAM events = 35
Control events = 30
0-10 years 0.98 (0.56-1.73), P = 0.955
Full follow-up HR 1.01 (0.62-1.65), P = 0.954
Number at risk
Control 42 19 17 14 0
Tamoxifen 48 22 19 16 0

Figure S3
Overall survival according to positive or negative PR status by different methods. Patients with available data for all methods for ER and PR were included (n=313). P-value by log-rank test, 5% significance level. a-b) cytosol-based method. c-d) IHC, e-f) gene expression, g-h) same result for all methods.
ER, Estrogen receptor; cytosol, cytosol-based method; IHC, immunohistochemistry; GEX, gene expression; PR, Progesterone receptor; TAM, Tamoxifen.
