# Supplementary material for: Hormone receptor mRNA and protein levels as predictors of premenopausal tamoxifen benefit
Source: Acta Oncol. 2024 Apr 8;63:19655. doi: 10.2340/1651-226X.2024.19655 (PMC11332536; doi:10.2340/1651-226X.2024.19655)
Supplement: Hormone receptor mRNA and protein levels as predictors of premenopausal tamoxifen benefit [file AO-63-19655-s2.pdf]

Supplementary material has been published as submitted. It has not been copyedited or typeset by Acta Oncologica.

Table S1. Combinations of ER and PR status by the three methods in 313 patients

	Cytosol	IHC	GEX*	Number of patients	
**ER**					
	+	+	+	178	
	+	+	-	0	Discordant evaluation No. (%) 51 (16)
	+	-	+	6	
	+	-	-	4	
	-	+	+	33	
	-	+	-	2	
	-	-	+	6	
	-	-	-	84	
				Total *313*	
**PR**					
	+	+	+	177	
	+	+	-	2	Discordant evaluation No. (%) 46 (15)
	+	-	+	12	
	+	-	-	11	
	-	+	+	13	
	-	+	-	1	
	-	-	+	7	
	-	-	-	90	
				Total *313*	

Patients with available data for all methods for ER and PR included (n=313).
Cytosol, cytosol-based method; *IHC*, immunohistochemistry; *GEX*, gene expression.
*Positive gene expression defined as ≥ 6 for *ESR1* and ≥ 4.5 for *PGR* on the normalized logarithmic scale presented from Nanostring.
